# Treating asthma with omega-3 fatty acids: where is the evidence? A systematic review

**DOI:** 10.1186/1472-6882-6-26

**Published:** 2006-07-19

**Authors:** J Reisman, HM Schachter, RE Dales, K Tran, K Kourad, D Barnes, M Sampson, A Morrison, I Gaboury, J Blackman

**Affiliations:** 1 Department of Pediatrics, Children's Hospital of Eastern Ontario, Ottawa, ON, Canada; 2 Chalmers Research Group, Children's Hospital of Eastern Ontario Research Institute, Ottawa, ON, Canada; 3 The Clinical Epidemiology Unit, Ottawa Health Research Institute, Ottawa Hospital and University of Ottawa, Ottawa, ON, Canada; 4 Canadian Coordinating Office for Health Technology Assessment, Ottawa, ON, Canada; 5 Complementary Medicine Program, University of Maryland, Baltimore, MD, USA

## Abstract

**Background:**

Considerable interest exists in the potential therapeutic value of dietary supplementation with the omega-3 fatty acids. Given the interplay between pro-inflammatory omega-6 fatty acids, and the less pro-inflammatory omega-3 fatty acids, it has been thought that the latter could play a key role in treating or preventing asthma. The purpose was to systematically review the scientific-medical literature in order to identify, appraise, and synthesize the evidence for possible treatment effects of omega-3 fatty acids in asthma.

**Methods:**

Medline, Premedline, Embase, Cochrane Central Register of Controlled Trials, CAB Health, and, Dissertation Abstracts were searched to April 2003. We included randomized controlled trials (RCT's) of subjects of any age that used any foods or extracts containing omega-3 fatty acids as treatment or prevention for asthma. Data included all asthma related outcomes, potential covariates, characteristics of the study, design, population, intervention/exposure, comparators, and co interventions.

**Results:**

Ten RCT's were found pertinent to the present report.

**Conclusion:**

Given the largely inconsistent picture within and across respiratory outcomes, it is impossible to determine whether or not omega-3 fatty acids are an efficacious adjuvant or monotherapy for children or adults. Based on this systematic review we recommend a large randomized controlled study of the effects of high-dose encapsulated omega-3 fatty acids on ventilatory and inflammatory measures of asthma controlling diet and other asthma risk factors. This review was limited because Meta-analysis was considered inappropriate due to missing data; poorly or heterogeneously defined populations, interventions, intervention-comparator combinations, and outcomes. In addition, small sample sizes made it impossible to meaningfully assess the impact on clinical outcomes of co-variables. Last, few significant effects were found.

## Background

Asthma is one of the most common chronic conditions, and affects both adults and children. Its prevalence has been noted to have increased significantly in Western nations with overall prevalence rates ranging from 7–15% [1]. The National Heart, Lung, and Blood Institute (NHLBI) has defined asthma as a chronic inflammatory disease of the airways[2].

The inflammatory response is complex, and involves a variety of inflammatory cell types including mast cells, alveolar macrophages, neutrophils, eosinophils, lymphocytes, platelets, and a variety of inflammatory mediators[3,4]. Over time, various therapeutic strategies have been developed to manage asthma, including the use of short acting beta-2 agonist bronchodilator medications as symptom relievers and anti-inflammatory preventer medications such as inhaled corticosteroids and oral leukotriene antagonists[2,5]. Given that the cause of airway inflammation can be multifactorial and involves a multitude of cell types and inflammatory mediators, the medications used to control inflammation may act at several different points in the inflammatory pathway[2,5]. Despite many therapeutic advances over the last thirty years, asthma continues to result in significant childhood and adult morbidity[5].

Given the rapidity of the increase in asthma's prevalence, environmental factors rather than increased genetic susceptibility are more likely to be responsible for this trend. Thus, there has been increased interest in investigating various environmental factors that may contribute to this illness, including diet. McKeever and Britton recently concluded that the trial evidence is less conclusive about the protective effects of fish oil intake on asthma and/or allergy than is the observational evidence[1]. There has been considerable interest in the potential therapeutic and protective value of dietary supplementation with the omega-3 fatty acids. Horrobin hypothesized that the low incidence of asthma in the northern aboriginal population stems from their consumption of large quantities of oily fish rich in omega-3 fatty acids[6]. Moreover, given the competitive interplay (e.g., for enzymes in the metabolic pathway and for positions in cell membranes) between pro-inflammatory omega-6 fatty acids, which may contribute to the cascade of events marking asthma, and the less pro-inflammatory omega-3 fatty acids, it has been thought that the latter could play a key role in treating or preventing asthma[7]. From this perspective, respiratory benefits might be attributable to changes in the status of mediators of inflammation such as leukotrienes and prostaglandins.

A systematic review was conducted to review the scientific-medical literature to identify, appraise and synthesize the evidence regarding the possible treatment and preventive value of omega-3 fatty acids in asthma[8]. This report describes the randomized controlled study (RCT) evidence concerning the efficacy of the treatment of asthma with omega-3 fatty acid supplementation in adult or pediatric populations. In addition to determining whether this intervention improves respiratory outcomes, its impact on the mediators of inflammation thought to be relevant to the pathogenesis of asthma is evaluated with this RCT evidence. Safety data are also presented.

## Methods

A comprehensive search for citations was conducted in April 2003 using six databases (MEDLINE, PreMEDLINE, EMBASE, Cochrane Central Register of Controlled Trials, Commonwealth Agriculture Bureau Health [CAB Health], and Dissertation Abstracts). All databases were searched via the Ovid interface using Search Strategy 1, except CAB Health which was searched through Silver Platter using Search Strategy 2 (See Additional file 1).

Searches were not restricted by language of publication, publication type, or study design except with the MeSH term "dietary fats," which was limited by study design to increase its specificity. Search elements included: scientific terms, with acronyms, as well as generic and trade names relating to the exposure and its sources (e.g., eicosapentaenoic acid [EPA]; MaxEPA^®^; fish oil); and relevant population terms (e.g., asthma; inflammation). Additional published or unpublished literature was sought through manual searches of reference lists of included studies and key review articles, and from the files of content experts. A final set of 1,010 unique references was identified and posted to an Internet-based software system for review.

Studies were considered to be relevant if they described human populations of any age, involved any type of study design, and investigated the use of any foods or extracts known to contain omega-3 fatty acids from any source (e.g., marine; plant) or of any type (e.g., EPA; alpha linolenic acid acid [ALA]) or dose, and used as a treatment or prevention. These eligibility criteria distinguish our work from that of a recent Cochrane review, which exclusively focused on the role of fish oil[9]. For the purposes of this report, however, only treatment RCT's are described because this research design is considered the gold standard for investigating questions of treatment efficacy or effectiveness[10,11]. Populations in treatment studies had to have received a formal diagnosis of asthma. Studies where an asthmatic response was experimentally induced in non-asthmatic populations were excluded. Methods had to have been employed in primary studies to identify the omega-3 fatty acid intervention/exposure. Studies investigating "polyunsaturated fatty acids" or specific diets were included if explicit reference was made to their omega-3 fatty acid content. A treatment study could assess respiratory outcomes, data concerning mediators of inflammation, or adverse effects. Forced expiratory volume in 1 second (FEV_1_) was selected as the primary outcome, given its status as a gold standard index of pulmonary function for asthma.

Two levels of screening for relevance, and two reviewers per level, were employed (bibliographic records, then full articles) following calibration exercises. Disagreements were resolved by forced consensus and if needed, third party intervention. Following a calibration exercise, three reviewers independently abstracted the contents of each included study using an electronic form. A second abstractor verified all data, which included outcomes (i.e., respiratory, mediators of inflammation, safety; discontinuations), potential covariates (e.g., fatty acid content of blood lipid biomarkers, tissue ratios of fatty acid during the investigative period), in addition to the characteristics of the report (e.g., publication status), study (e.g., sample size; design), population, intervention/exposure (e.g. omega-3 fatty acid types and sources; intervention length), comparators (e.g., placebo), and co interventions (e.g. asthma medications, omega-6 fatty acids). Dual-assessor evaluations of RCTs' quality employed Jadad's validated items concerning randomization, double blinding, and the reporting of withdrawals and dropouts,[12] and b) Schulz's validated question concerning the adequacy of allocation concealment[13]. A total Jadad score below 3 (out of 5) indicates low study quality. Select items from a third validated instrument permitted the assessment of the quality of other designs, which are excluded from the present report[14]. The applicability, or generalizability, of a study to a broad-based North American population was determined by the degree to which the study population included asthma patients who were otherwise "healthy," potentially exhibiting asthma concomitants (e.g., atopy), representing a somewhat broad demographic spectrum (e.g., sex, ethnicity), living a "typical" North American lifestyle, possibly receiving "typical" asthma treatment, and failing to exhibit major co morbidity.

## Results

Of the 1,010 bibliographic records entered into the initial screening for relevance, 851 were excluded. All but 5 of the remaining 159 reports were then retrieved and subjected to a more rigorous relevance assessment. The second relevance screening then excluded 122 reports. In total, 31 reports, describing 26 unique studies, were deemed relevant for the systematic review of treatment or prevention evidence, with 5 studies each described by two reports. Nineteen treatment studies were identified, ten of which were RCTs pertinent to the present report (Table 1) [15-24]. Two treatment RCTs employed a crossover design[15,21].

**Table 1:** RCT Evidence of Omega-3 Fatty Acids to Improve Respiratory Outcomes in Asthma

	**Study groups^1^**			
			
**Author, Year, Location: Length & Design**	**Group 1 (n)/Group 4 (n)**	**Group 2 (n)/Group 3 (n)**	**Jadad Total Quality/Allocation Concealment (Internal Validity)**	**Applicability**
**ADULTS**				

Arm, 1988[18] England: 10-wk parallel RCT	3.2 g/d EPA + 2.2 g/d DHA (n = NR)	Olive oil placebo (dose: NR) (n = NR)	4/Unclear	III
Emelyanov, 2002[23] Russia: 8-wk parallel RCT	200 mg/d EPA/DHA + 400 mg/d olive oil (n = 23)	600 mg/d olive oil placebo (n = 23)	5/Unclear	III
Kirsch, 1988[22] USA: 8-wk parallel RCT	4.0 g/d EPA ethyl ester (+ trace DHA) (n = 6)	0.1 g/d EPA ethyl ester (+ trace DHA) (n = 6)	3/Unclear	I
McDonald, 1991[15] Australia: 10-wk crossover RCT	2.7 g/d EPA + 1.8 g/d DHA (n = 15)	15 g/d olive oil placebo (n = 15)	3/Unclear	III
Okamoto, 2000[19] Japan: 4-wk RCT	10–20 g/d perilla seed oil (ALA: NR) (n = 7)	10–20 g/d corn oil (n = 7)	2/Unclear	III
Stenius-Aarniala, 1989[21] Finland: 10-wk crossover RCT	20 mL/d fish oil (EPA+DHA: NR) (n = 36)	20 mL/d olive oil (n = 36)/20 mL/d evening primrose oil (n-6) (n = 36)	2/Unclear	III
Thien, 1993[20] England: 26-wk parallel RCT	3.2 g/d EPA + 2.2 g/d DHA (n = NR)	Olive oil placebo (dose: NR) (n = NR)	4/Unclear	III

**CHILDREN**				

Hodge, 1998[17] Australia: 26-wk parallel RCT	0.72 g/d EPA 0.48 g/d DHA + ALA (dose: NR) via canola diet (n = NR)	n-6: 1.8 g/d safflower oil + 1.8 g/d palm oil + 0.4 g/d olive oil + sunflower diet (dose: NR) (n = NR)	3/Unclear	III
Nagakura, 2000[16] Japan: 10-mo parallel RCT	17.0–26.8 mg/kg/d EPA; 7.3–11.5 mg/kg/d DHA (300 mg/d fish oil) (n = 15)	300 mg/d olive oil placebo (n = 15)	4/Unclear	III

**AGE NOT REPORTED**				

Dry, 1991[24] France: 9-mo parallel RCT	1.0 g/d EPA+DHA (n = NR)	"Placebo" (type & dose: NR) (n = NR)	2/Unclear	X

^1^Proceeding from highest omega-3, or lowest omega-6/omega-3, fatty acid content of intervention; n-6 = omega-6 FAs; ALA = alpha linolenic acid; DHA = docosahexaenoic acid; EPA = eicosapentaenoic acid; Length = intervention length; Design = research design; n = sample size; pts = study participants; NR = not reported; wk = week(s); mo = month; RCT = randomized controlled trial; Jadad total quality score = /5; Applicability I = greatest applicability to North American population of asthma patients; Applicability II = somewhat narrow applicability; Applicability III = very narrow applicability; Applicability X = insufficient data to determine applicability

Nine treatment studies employed other designs, and seven studies investigated the possible primary preventive value of omega-3 fatty acid intake. The ten treatment RCTs were described in 13 reports. Hodge et al.'s pediatric trial results[17] were first disseminated as an abstract [25] Kirsch et al.'s adult treatment RCT had its clinical outcome data reported in one publication[22] and its mediators of inflammation data in another[26]. The lead author (Jonathan Arm) of two study reports with overlapping data recommended to us that, to avoid entering duplicate data into our review, we should consult the first report[18] for all data other than allergen-challenge results, which should be obtained from the second document[27]. To simplify matters, only one document per study is referred to in this report's text or table. Only one treatment RCT was not described by at least one published report[15]. All treatment RCT reports were published in English.

Two trials exclusively randomized children,[16,17] one included both older adolescents and adults,[18] and the remainder randomized adults [15,19-24]. One report did not identify the age of its participants[24]. Given the differences in the clinical pictures of adult and (especially young) children's asthma, results obtained from studies enrolling the different populations were not combined.

Given the largely inconsistent picture within and across respiratory outcomes, it is impossible to conclude one way or another whether omega-3 fatty acids are an efficacious adjuvant or monotherapy in improving respiratory outcomes in asthmatic adults or children. This is perhaps best illustrated by what was observed with respect to the primary outcome variable, FEV_1_.

The adult RCTs revealed a somewhat contradictory picture of efficacy with respect to FEV_1_. One very small adult study conducted by Okamoto et al.(n = 14), which compared "uncontrolled dosing" of perilla seed oil (i.e., pourable oil) and similarly uncontrolled dosing of corn oil over a short intervention period (4 weeks), reported a significant positive clinical effect[19]. However, two RCTs each observed no benefit. Kirsch et al.'s RCT compared high and low doses of EPA ethyl ester over 8 weeks in a small study (n = 12),[22] whereas Emelyanov investigated the impact of low dose EPA+DHA (versus an olive oil placebo) over 8 weeks in the systematic review's highest quality RCT[23]. The latter study also randomized the second largest sample population (n = 46) included in our evidence review. Investigation of additional clinical outcomes for adults revealed that a) for AM peak expiratory flow (PEF), two studies reported a significant benefit, [19,23]while three reported no benefit,[18,20,21,23] whereas b) five studies failed to observe a significant benefit for PM PEF. Inconsistent results were observed with respect to each of three self-reported outcomes, including bronchodilator use, asthma symptom scores and asthma severity ratings. With regard to pediatric studies, one RCT observed no benefit with respect to FEV_1_[17]. There were too few pediatric studies with which to observe any consistent patterns of result for any outcome.

No study, either involving adults or children, received an allocation concealment rating other than "Unclear." Only two adult studies,[19,21] in addition to the RCT for which age data were not provided,[24] were found to exhibit low (total Jadad score: <3) study quality. There was very limited generalizability to a broad spectrum North American population of asthmatics for all but one of the studies, which was conducted in the United States[22].

Several key observations made it inappropriate to consider conducting meta-analysis, including missing data (e.g., sample sizes; type and dose of omega-3 fatty acids; types and doses of concurrent asthma medication with the potential to confound results; placebo contents), poorly or heterogeneously defined populations (e.g., age, exact diagnosis, asthma severity, asthma triggers or concomitants), interventions (e.g., sources, types i.e. pharmaceutical versus health-food store grade fish oil, doses, and the degree of control over dosing of omega-3 fatty acids [capsules versus pourable oils]), intervention-comparator combinations, and outcomes. Some of the published studies used peak flow rate as their primary outcome measure, rather than the more reliable FEV_1_. In addition to typically small sample sizes, these study limitations also made it impossible to meaningfully assess, even qualitatively, the impact on clinical outcomes of co variables such as the source, type and dose of omega-3 fatty acids. This objective was further complicated by the fact that few significant effects were found. These study limitations likewise made it impossible to ascertain, based on four adult trials[18,19,21,22] and one pediatric RCT,[17] whether omega-3 fatty acids consistently and positively influence the lipid mediators of inflammation in ways congruent with the biological model implicating the lipoxygenase and cyclooxoygenase pathways in asthma.

Six of eight adult RCTs[15,18,20-23] and the two pediatric trials[16,17] each reported safety data. Most of the adverse events were related to the capsule delivery of oils, rather than to the oils per se[15,16,18,20]. On several occasions, difficulty swallowing capsules led to a withdrawal from the study. In two studies, participants may have had difficulties swallowing 18 capsules of oil per day, yet these difficulties were not reported[18,20]. The single most serious reaction noted was an undefined number of episodes of nausea and vomiting after ingesting fish oil capsules, which in turn led to study withdrawal[20]. There were unspecified numbers of children and adults who experienced mild gastrointestinal upset, yet not all of these individuals had received the omega-3 fatty acid exposure. Fishy tasting eructations were reported by some study participants.

## Discussion

Although the use of omega-3 fatty acid supplementation is popular and its purported beneficial effects are biologically plausible, the existing body of evidence from clinical trials does not provide strong support for its clinical effectiveness. The adverse effects of nausea and abdominal discomfort do however appear to be consistent across studies and appear to be dose-related. There is no consistent evidence supporting an improvement in objective measures of ventilatory lung function in adults with the majority of studies of FEV_1_[19,22,23] and PEF [18-21,23] failing to find a benefit of omega-3 fatty acid supplementation. More often than not, other outcomes such as asthma symptoms, clinical severity scores, and 'rescue inhaler use' did not significantly improve. No RCT's, which investigated non-marine sources of omega-3 fatty acids, were found. In only the study by Hodge et al, was dietary manipulation of n-3 PUFA performed as part of the treatment phase[17]. While potential changes to mediator generation may not be as marked with dietary manipulation as compared to supplement administration, an acceptable food-based approach may be better tolerated by individuals in the long run, should benefit be ultimately proven.

Since this systematic review has been completed, an additional study has been published that would have met our eligibility criteria[28]. Mickleborough and co-workers have studied the protective effect of fish oil supplementation on exercise-induced bronchoconstriction (EIB) in 16 adult asthmatic patients. Those whose diet was supplemented with fish oil had improved pulmonary function to below the diagnostic EIB threshold in post-exercise FEV1, a reduced fall in FEV1 15 minutes post exercise, reduced bronchodilator requirement, and a reduction of a variety of inflammatory mediators. The authors concluded that fish oil might represent a possible non-pharmacologic therapeutic strategy for asthmatic subjects with EIB. However, this observation does not alter our own overall conclusions[28].

The inability, due to strong inter-study clinical heterogeneity (i.e., populations; interventions; outcomes), to combine the results in a formal meta-analysis reduces the effective sample size from which to discern benefits or harm. It is not possible to determine if differences in clinical benefit between studies was caused by differences in characteristics of the study groups, interventions or outcomes, or simply due to chance. To clarify this, large randomized clinical trials are needed. A daily intervention of at least 3 grams of omega-3 fatty acids, which is considered a high adult dose, may well be required. Its source (e.g. marine, plant) and constituents (i.e., ALA, EPA, EPA+DHA) need to be well defined, although it is as yet unclear which combinations of omega-3 fatty acids should be employed. Not only should omega-3 supplementation be studied, but also dietary manipulation. Duration of intervention, minimal effective dosage, and formulation will need to be determined. Outcomes should reflect the dimensions of asthma; symptoms, use of rescue inhaler, ventilatory function, variability in function, and acute exacerbations. Exhaled nitric oxide is a newer measure of asthma inflammation. Exhaled breath condensate could be collected and tested for leukotrienes and prostaglandins, mediators of inflammation potentially influenced by omega-3 fatty acids. Induced sputum techniques could also be utilized to assess airway inflammatory cell and mediator characteristics.

Many sources of influencing and potentially confounding variables need to be controlled in the study design, measured during the study and perhaps adjusted for in the analysis.

Age and gender influence asthma severity and prevalence of atopy. Co-existing anti-inflammatory medications may overwhelm a similar but lesser effect of omega-3 fatty acids. Dietary omega-6 fatty acids need to be controlled for since they may compete with omega-3 fatty acids for enzymes and positions in cell membranes and neutralize the effect. Using encapsulated rather than pourable oils would add precision to the dose of omega-3 fatty acid supplementation. A relatively long follow-up period of one or two years would facilitate the assessment of exacerbation rates. Changes in exposure to indoor allergens and irritants may also influence asthma control. In conclusion, given the data available to date, and despite an acceptable safety profile, a recommendation as to whether omega-3 fatty acids are a viable treatment modality is at present unresolved. Recommendations for future research follow directly from observations of the problems and limitations observed in the included studies.

## Conclusion

Given the largely inconsistent picture within and across respiratory outcomes, it is impossible to determine whether or not omega-3 fatty acids are an efficacious adjuvant or monotherapy for children or adults. Based on this systematic review we recommend a large randomized controlled study of the effects of high-dose encapsulated omega-3 fatty acids on ventilatory and inflammatory measures of asthma controlling diet and other asthma risk factors. This review was limited because Meta-analysis was considered inappropriate due to missing data; poorly or heterogeneously defined populations, interventions, intervention-comparator combinations, and outcomes. In addition, small sample sizes made it impossible to meaningfully assess the impact on clinical outcomes of co-variables. Last, few significant effects were found.

## Competing interests

Financial competing interests

Authors of this manuscript (and corresponding review) have not received any reimbursements fees, funding or salary form organizations that may in anyway gain or lose financially from the publication of this manuscript in the past five years prior to start of the corresponding review.

Authors do not hold any stocks or shares in an organization that may in any way gain or lose financially from the publication of this manuscript.

Authors do not hold or are currently applying for any patents relating to the content of the manuscript, nor they have received reimbursements, fees, funding, or salary from an organization that holds or has applied for patents relating to the content of the manuscript.

Non-financial competing interests

Authors have no non-financial competing interests (political, personal, religious, ideological, academic, intellectual, commercial or any other) to declare in relation to this manuscript.

## Authors' contributions

JR: led the conceptualization of the review and the manuscript and provided content expertise

HS: coordinated the systematic review and lead the conceptual design of the review and manuscript, screened on all levels, verified data, was the primary author of corresponding review, and also drafted the manuscript

RED: collaborated in conceptualizing the review elements and provided content expertise

KT: collaborated in conceptualizing the review elements and provided content expertise

KK: collaborated in conceptualizing the review elements and provided content expertise

DB: collaborated in conceptualizing the review elements and provided content expertise

MS: specialized search for the corresponding review

AM: technical information specialist assistance

IG: statistical expertise, meta analysis of the corresponding review

JB: collaborated in conceptualizing the review elements and provided content expertise

CG: participated in coordination, and data abstraction

All authors have read and approved this manuscript.

## Pre-publication history

The pre-publication history for this paper can be accessed here:



## Supplementary Material

Additional File 1 Search Strategies. Search strategy 1 & 2, strategies used to search various databases for this review. Click here for file
