# Supplementary material for: Treating asthma with omega-3 fatty acids: where is the evidence? A systematic review
Source: BMC Complement Altern Med. 2006 Jul 19;6:26. doi: 10.1186/1472-6882-6-26 (PMC1550729; doi:10.1186/1472-6882-6-26)
Supplement: Additional File 1 — Search Strategies. Search strategy 1 & 2, strategies used to search various databases for this review. [file 1472-6882-6-26-S1.doc]

## Search Strategies

# Search Strategy 1

1. exp Asthma/

2. Bronchial hyperreactivity/

3. asthma$.mp.

4. wheez$.mp.

5. respiratory sounds/

6. exp LEUKOTRIENES/

7. leukotrien$.mp.

8. (lung$ or pulmon$ or respirat$).mp. [mp=title, abstract, cas registry/ec number word, mesh subject heading]

9. exp INFLAMMATION/

10. exp Inflammation Mediators/

11. inflammat$.mp.

12. 8 and (or/9-11)

13. or/1-7,12

14. exp fatty acids, omega-3/

15. fatty acids, essential/

16. Dietary Fats, Unsaturated/

17. linolenic acids/

18. exp fish oils/

19. (n 3 fatty acid$ or omega 3).tw.

20. docosahexa?noic.tw,hw,rw.

21. eicosapenta?noic.tw,hw,rw.

22. alpha linolenic.tw,hw,rw.

23. (linolenate or cervonic or timnodonic).tw,hw,rw.

24. menhaden oil$.tw,hw,rw.

25. (mediterranean adj diet$).tw.

26. ((flax or flaxseed or flax seed or linseed or rape seed or rapeseed or canola or soy or soybean or walnut or mustard seed) adj2 oil$).tw.

27. (walnut$ or butternut$ or soybean$ or pumpkin seed$).tw.

28. (fish adj2 oil$).tw.

29. (cod liver oil$ or marine oil$ or marine fat$).tw.

30. (salmon or mackerel or herring or tuna or halibut or seal or seaweed or anchov$).tw.

31. (fish consumption or fish intake or (fish adj2 diet$)).tw.

32. diet$ fatty acid$.tw.

33. or/14-32

34. dietary fats/

35. (randomized controlled trial or clinical trial or controlled clinical trial or evaluation studies or multicenter study).pt.

36. random$.tw.

37. exp clinical trials/ or evaluation studies/

38. follow-up studies/ or prospective studies/

39. or/35-38

40. 34 and 39

41. (Ropufa or MaxEPA or Omacor or Efamed or ResQ or Epagis or Almarin or Coromega).tw.

42. (omega 3 or n 3).mp.

43. (polyunsaturated fat$ or pufa or dha or epa or long chain or longchain or lc$).mp.

44. 42 and 43

45. 33 or 40 or 41 or 44

46. 13 and 45

47. limit 46 to human

# Search Strategy 2

#1 omega 3

#2 ("essential-fatty-acids" in SU) or ("linolenic-acid" in SU)

#3 ("docosahexaenoic-acid" in SU) or ("eicosapentaenoic-acid" in SU)

#4 explode "plant-oils" in SU

#5 explode "fish-oils" in SU

#6 "fish-consumption" in SU

#7 "polyenoic-fatty-acids" in SU

#8 "polyunsaturated-fats" in SU

#9 "dietary-fat" in SU

#10 (n 3 fatty acid* or omega 3) in ti,ab,id

#11 (docosahexanoic or docosahexaenoic) in ti,ab,id

#12 (eicosapentanoic or eicosapentaenoic) in ti,ab,id

#13 (alpha linolenic)in ti,ab,id

#14 (linolenate or cervonic or timnodonic) in ti,ab,id

#15 (mediterranean diet) in ti,ab,id

#16 ((flax or flaxseed or flax seed or linseed or rape seed or rapeseed or canola or soy or soybean or walnut or mustard seed or menhaden) and oil*) in ti,ab,id

#17 (walnut* or butternut* or soybean* or pumpkin seed*) in ti,ab,id

#18 (fish oil* or cod liver oil* or marine oil* or marine fat*) in ti,ab,id

#19 (salmon or mackerel or herring or tuna or halibut or seal or seaweed or anchov*) in ti,ab,id

#20 (fish consumption or fish intake) in ti,ab,id

#21 (diet* fatty acid*) in ti,ab,id

#22 (ropufa or maxepa or omacor or efamed or resq or epagis or almarin or coromega) in ti,ab,id

#23 ((omega 3 or n 3) and (polyunsaturated fat* or pufa or dha or epa or long chain or longchain or lc*)) in ti,ab,id

#24 "long-chain-fatty-acids" in SU

#25 (fish and diet) in ti,ab,id

#26 ((fish and diet) in ti,ab,id) or ("long-chain-fatty-acids" in SU) or (((omega 3 or n 3) and (polyunsaturated fat* or pufa or dha or epa or long chain or longchain or lc*)) in ti,ab,id) or ((ropufa or maxepa or omacor or efamed or resq or epagis or almarin or coromega) in ti,ab,id) or ((docosahexanoic or docosahexaenoic) in ti,ab,id) or ((n 3 fatty acid* or omega 3) in ti,ab,id) or ("dietary-fat" in SU) or ("polyunsaturated-fats" in SU) or ("polyenoic-fatty-acids" in SU) or ("fish-consumption" in SU) or (explode "fish-oils" in SU) or (explode "plant-oils" in SU) or (("docosahexaenoic-acid" in SU) or ("eicosapentaenoic-acid" in SU)) or (("essential-fatty-acids" in SU) or ("linolenic-acid" in SU)) or ((diet* fatty acid*) in ti,ab,id) or ((fish consumption or fish intake) in ti,ab,id) or ((salmon or mackerel or herring or tuna or halibut or seal or seaweed or anchov*) in ti,ab,id) or ((fish oil* or cod liver oil* or marine oil* or marine fat*) in ti,ab,id) or ((walnut* or butternut* or soybean* or pumpkin seed*) in ti,ab,id) or (((flax or flaxseed or flax seed or linseed or rape seed or rapeseed or canola or soy or soybean or walnut or mustard seed or menhaden) and oil*) in ti,ab,id) or ((mediterranean diet) in ti,ab,id) or ((linolenate or cervonic or timnodonic) in ti,ab,id) or ((alpha linolenic)in ti,ab,id) or ((eicosapentanoic or eicosapentaenoic) in ti,ab,id)

#27 Bronchial hyperreactiv*

#28 Asthma*

#29 Wheez*

#30 Leukotrien*238

#31 explode "leukotrienes-" in SU

#32 "respiratory-hypersensitivity" in SU

#33 explode "asthma-" in SU

#34 Lung* or pulmon* or respirat*

#35 explode "inflammation-" in SU

#36 Inflammat*

#37 #34 and (#35 or #36)

#38 (explode "leukotrienes-" in SU) or (Leukotrien*) or (Wheez*) or (Asthma*) or (#34 and (#35 or #36)) or (Bronchial hyperreactiv*) or (explode "asthma-" in SU) or ("respiratory-hypersensitivity" in SU)

#39 #26 and #38

#40 "man" in od

#41 #40 and #39
